# Broadly Protective Neuraminidase-Based Influenza Vaccines and Monoclonal Antibodies: Target Epitopes and Mechanisms of Action

**DOI:** 10.3390/v15010200

**Published:** 2023-01-10

**Authors:** Nada Abbadi, Jarrod J. Mousa

**Affiliations:** 1 Center for Vaccines and Immunology, College of Veterinary Medicine, University of Georgia, Athens, GA 30602, USA; 2 Department of Infectious Diseases, College of Veterinary Medicine, University of Georgia, Athens, GA 30602, USA; 3 Department of Biochemistry and Molecular Biology, Franklin College of Arts and Sciences, University of Georgia, Athens, GA 30602, USA

**Keywords:** influenza, neuraminidase, universal influenza vaccine, antibodies, monoclonal antibodies

## Abstract

Neuraminidase (NA) is an important surface protein on influenza virions, playing an essential role in the viral life cycle and being a key target of the immune system. Despite the importance of NA-based immunity, current vaccines are focused on the hemagglutinin (HA) protein as the target for protective antibodies, and the amount of NA is not standardized in virion-based vaccines. Antibodies targeting NA are predominantly protective, reducing infection severity and viral shedding. Recently, NA-specific monoclonal antibodies have been characterized, and their target epitopes have been identified. This review summarizes the characteristics of NA, NA-specific antibodies, the mechanism of NA inhibition, and the recent efforts towards developing NA-based and NA-incorporating influenza vaccines.

## 1. Introduction

Influenza is an acute respiratory infection that causes significant global morbidity and mortality annually, as well as a large economic burden [1]. In addition, influenza pandemics remain a threat and can occur at irregular and unpredictable intervals, claiming millions of lives [2]. Influenza virus has two surface glycoproteins with large extracellular domains: hemagglutinin (HA) and neuraminidase (NA) [3], which play essential roles in influenza infection. HA is more abundant on the viral surface than NA [4], and the immune response to influenza infection or vaccination is mainly targeted towards the HA protein [5,6]. However, early studies demonstrated that NA-specific antibodies are protective and successful in reducing virus shedding and infection severity [7]. NA has been established as a primary drug target for the prophylaxis and treatment of influenza infection. NA inhibitors such as oseltamivir, zanamivir or peramivir are available for use in influenza infection treatment [8], but they provide the most benefit when given within 24–48 h after infection [9]. In some cases, amino acid substitutions affecting the NA enzymatic site may occur, leading to drug resistance [10]. However, the rate of drug resistance is generally low, with <3.5% resistance to oseltamivir and <1% resistance to zanamivir [11].

The primary mechanism of protection against influenza infection is vaccination. Live-attenuated, split-inactivated, and recombinant protein platforms are all currently used in influenza vaccines [12]. Current seasonal influenza vaccines induce narrow strain-specific immune responses, and the effectiveness can be variable depending on how well the vaccine strains match circulating strains [13]. Therefore, broadly protective universal influenza virus vaccines are needed. This article will review recent progress in our understanding of NA and NA-specific antibodies in efforts toward the development of universal influenza vaccines.

## 2. The Role of Neuraminidase in the Influenza Virus Life Cycle

Viruses from the family Orthomyxoviridae are enveloped viruses characterized by having eight segmented, negative-sense RNA genomes [14]. There are currently four types of influenza viruses: A, B, C, and D [15]. Influenza A viruses (IAV) are further subtyped according to their surface glycoproteins, HA and NA [16]. HA is divided into 18 subtypes (H1–H18), while NA is divided into 11 subtypes (N1–N11) [17]. Only two of these subtypes are currently endemic in humans (H1N1 and H3N2) [18]. Among the 18 subtypes of HA, they are divided into two groups: Group 1 includes H1, H2, H5, H6, H8, H9, H11, H12, H13, and H16, while group 2 contains H3, H4, H7, H10, H14, and H15 [19]. The 11 subtypes of NA are classified into three groups: group 1 contains N1, N4, N5, and N8; group 2 includes N2, N3, N6, N7, and N9; and group 3 contains NA from influenza B viruses (IBV) [20]. From the 11 NA subtypes, only the N1 and N2 subtypes have caused epidemics in humans [21]. Due to the errors that could occur with RNA dependent RNA polymerases in this replication cycle, influenza viruses are able to effectively evade the immune system [22]. Therefore, it is difficult to develop long lasting immunity to these viruses. Current seasonal influenza vaccines mainly target HA [23]. However, there are other potential targets that play an essential role in this life cycle, including NA and M2 [23].

The influenza life cycle begins with HA attachment to sialic-acid (SA) receptors on the host cell surface [24]. SA is found on the surface of many cells located in the respiratory tract, such as epithelial cells, dendritic cells, and alveolar macrophages [17]. The type of linkage that connects SA to the oligosaccharide of the receptor molecule plays a role in the efficiency of HA binding to SA. Human-tropic influenza viruses typically bind to SA attached to galactose by an α2,6 linkage, while avian-tropic viruses prefer α2,3 linked SA molecules [25]. Following the infection cycle, newly assembled viruses remain attached to SA receptors on host cells by HA. The virus is then released by the sialidase activity of NA, which cleaves SA, releasing the virus from host cells [23]. The sialidase activity of NA also facilitates the removal of SA residues from the viral surface, preventing the aggregation of newly assembled viruses near the cell surface, which improves infectivity [26]. 

## 3. Neuraminidase Structure and Function

NA is assembled as a homotetramer of approximately 240 kDa in size and is anchored in the envelope of the virus [27]. Each of the monomers contains four distinct structural domains: the head, the stalk, the transmembrane domain, and the cytoplasmic tail [20]. The sequence of the cytoplasmic tail is nearly 100% conserved across all IAV subtypes, and it plays an important role during NA transport and incorporation [20]. The N-terminal hydrophobic transmembrane domain contains a variable sequence of amino acids and provides signals for translocation to the membrane [28]. The length of the stalk domains impacts the NA enzymatic activity and virulence [29]. In addition, glycosylation can also impact virulence and infectivity [30]. Studies have shown that loss of glycosylation on both HA and NA makes the viruses more virulent [30,31]. The head domain of NA consists of a mushroom-shaped structure comprising four monomers [32]. It is responsible for cleaving SA residues and releasing the virus from infected host cells, as well as preventing viral aggregation near the cell surface [33]. The tetrameric conformation of NA is essential for enzymatic activity and for the elicitation of protective antibodies [34]. While the primary function of NA is the release of newly budded virions from host cells [35], NA also plays a role in virus entry, which can be accomplished either by direct receptor binding, complementing the receptor-binding function of HA, or by helping release virions bound to sialylated “decoy” receptors like mucins, facilitating virus access to the cell surface [36]. As HA and NA display antagonistic functions [37], a balance is maintained between the activities of both proteins to ensure efficient entry and release of influenza viruses.

## 4. Immune Response to NA

**Figure 1:**
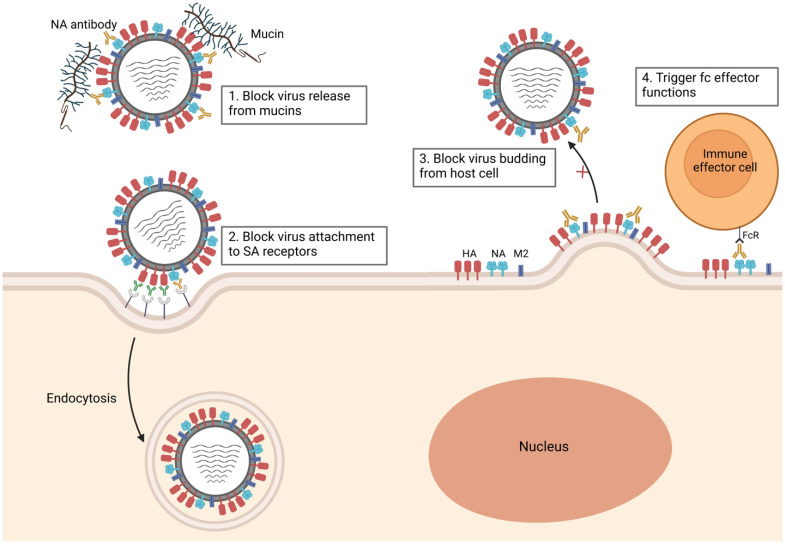
Mechanism of neuraminidase (NA) inhibition by NA-specific antibodies. (**1**) NA-specific antibodies prevent viral release from mucins. (**2**) Virus attachment to sialic acid receptors (SA) is blocked by NA-specific antibodies as well as hemagglutinin (HA) antibodies. (**3**) Virus release from the host cell is blocked by NA antibodies. (**4**) NA-specific antibodies trigger Fc effector functions such as antibody-dependent cellular cytotoxicity (ADCC) and antibody-dependent phagocytosis (ADP) through binding with Fc receptors on immune effector cells (natural killer (NK) cells, macrophages, and dendritic cells).

The constant immune pressure on influenza viruses leads to the generation of new strains with minor changes in their structure in a process called antigenic drift, rendering pre-existing antibodies potentially less effective [38]. Consequently, the seasonal influenza vaccine must be updated annually to match the circulating strain [39]. Anti-HA-specific antibodies can block an influenza infection by blocking the viral attachment to the host cell surface [23]. In some cases, anti-HA antibodies were found to also block the action of NA by blocking virus binding to the surface-bound NA substrate or by sterically inhibiting NA access to the substrates [40]. The breadth of the antibody response to an influenza infection greatly depends on the individual’s previous exposure to the virus [41]. Therefore, adults usually show a broader antibody response than children, who have limited exposures [23]. While NA also undergoes antigenic drift similar to that of HA, the process occurs much slower and independently of HA antigenic drift [42,43]. Therefore, NA is considered a promising target for a universal influenza vaccine. Following vaccination with current virion-based vaccines, the immune response primarily targets HA, with less NA antibodies elicited [44]. Natural infection usually triggers a more balanced immune response towards both HA and NA [6]. High seroconversion rates were measured against HA and NA by enzyme-linked immunosorbent assay (ELISA) in recent studies [45]. H1N1 pandemic influenza virus-infected patients showed that seroconversion to NA was detected at day 7 and peaked at day 28, and antibodies began to decline at day 90 [46]. It has been shown in mice that NA-inhibiting antibodies are associated with a reduction in lung viral titers [47]. Additionally, isolated human monoclonal anti-NA antibodies were shown to reduce airborne transmission of the human influenza A virus when the antibodies were administered post-infection to guinea pigs [48]. Anti-NA antibodies can be protective by inhibiting the enzymatic activity of the virus by direct binding or steric hindrance of the active site [49]. Since NA antibodies act at the late stages of the viral life cycle, they are predominantly non-neutralizing. Therefore, virus titer is usually not affected in a plaque reduction assay, but plaque diameter is significantly reduced in the presence of NA antibodies [50,51]. In addition to preventing viral release, NA-specific antibodies can function through multiple mechanisms (Figure 1). Antibodies against NA can prevent viral release from mucins, inhibiting viral access to the cell surface, and have been shown to inhibit viral attachment to sialic receptors and entry similar to HA-specific antibodies [36]. Additionally, some NA-specific antibodies can function through the Fc domain, which interacts with Fcγ receptors (FcγRs) expressed on macrophages, dendritic cells (DCs), and natural killer (NK) cells [52,53]. Fc–FcγRs interactions can lead to antibody-dependent cellular cytotoxicity (ADCC) mediated by natural killer (NK) cells. Activated NK and macrophages produce the antiviral cytokine IFN-γ and degranulate or phagocytose infected cells, resulting in clearance of virus-infected cells [52,53]. Recent studies revealed that NA inhibition (NAI) titers correlated with protection against influenza virus, reducing symptoms and resulting in decreased viral shedding [54,55,56]. In some cases, NAI titers are better correlates of protection than HAI titers, which are generally considered the gold standard [57,58]. Several studies showed that antibody responses to vaccination are short-lived compared to antibody responses to natural infection and that vaccination responses may decline within a given influenza season [59,60,61].

## 5. NA mAbs and Target Epitopes

**Figure 2:**
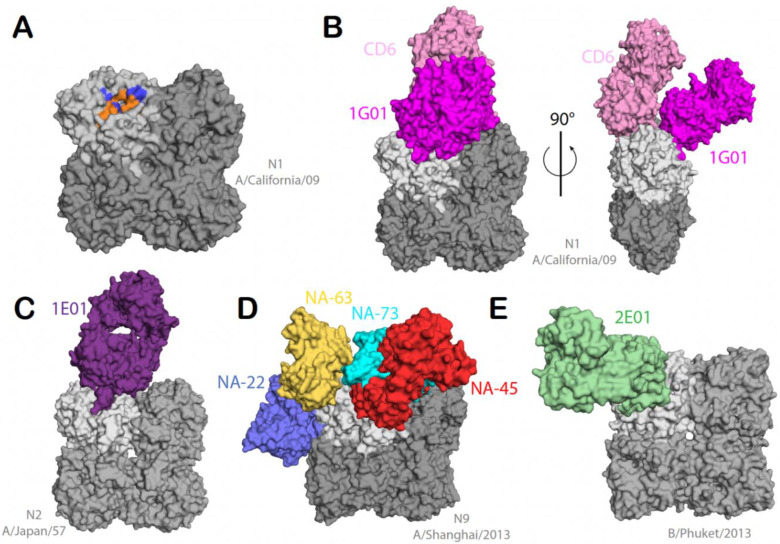
Neuraminidase antibodies target epitopes. (**A**) The NA active site is conserved across subtypes. Orange shows eight functional residues on the active site, and blue shows 11 framework residues on one NA protomer. (**B**) NA protein from H1N1 subtype A/California/04/2009 (grey) in complex with 1G01 Fab (magenta) (PDB: 6Q23) and CD6 Fab (pink) (PDB: 4QNP). (**C**) NA protein from H2N2 subtype A/Japan/305/1957 (grey) in complex with 1E01 Fab (purple) (PDB: 6Q20). (**D**) NA protein from H7N9 subtype A/Shanghai/2/2013 (grey) in complex with NA-45 Fab (red) (PDB: 6PZE), NA-73 (cyan) (PDB: 6PZY), NA-63 (yellow) (PDB:6U02), and NA-22 (blue) (PDB: 6PZW). (**E**) NA protein from influenza B/Phuket/3073/2013 (grey) in complex with 2E01 Fab (green) (PDB: 6V4O).

Compared to HA, less is known about antibody epitopes and antigenic sites on NA. Characterizing these epitopes will help in understating the antigenic drift of NA and future universal vaccine development. In order to characterize and identify NA antigenic sites, several studies were carried out using mAbs and/or recombinant NA proteins [49,62,63,64]. The active site of NA consists of 8 functional residues (R118, D151, R152, R224, E276, R292, R371, and Y406) and 11 framework residues (E119, R165, W178, S179, D198, I222, E227, H274, E277, N294, and E425) (Figure 2A). These residues are highly conserved among all NA subtypes [26]. A universally conserved linear epitope between amino acids 222 and 230 located near the NA active site was identified by Gravel et al. [65]. mAbs to this epitope showed binding to nine subtypes of NA proteins [65]. A follow up study showed that amino acids I222 and E227, in particular, were essential for binding with these mAbs [66]. The NA inhibitory effect shown by these mAbs was eliminated against I222A and E227A mutants [66]. The residue D151 is located at the edge of the NA active site, and it is known to be sensitive to oseltamivir, which suggests that this residue is critical and involved in the catalytic activity of NA [67]. In vitro, the human mAb 1G01 was isolated from H3N2 infected individuals and binds to and inhibits the activity of several group 1 and group 2 NAs as well as NAs from both influenza B virus antigenic lineages [49]. mAbs 1G01 and 1E01 were shown to inhibit NA activity by occupying the catalytic site via a long CDR H3 loop, as shown by co-crystal structure analysis [49] (Figure 2B,C). Structural analysis of an N1 mAb CD6 showed that the antibody binds to two neighboring NA monomers [68] (Figure 2B). Targeting this epitope, which consists of 30 amino acids located on an NA dimer, with CD6 mAb protected mice from a lethal influenza infection by inhibiting NA enzymatic activity via steric hindrance [68]. Anti-N9 mAbs NA-45, NA-22, NA-63, and NA-73 targeting different epitopes on the N9 protein were isolated from a healthy subject vaccinated with a monovalent inactivated A/Shanghai/02/2013 (Sh2) H7N9 vaccine [69] (Figure 2D). NA-45 mAb recognized the conserved NA epitopes shown in (Figure 2A) and inhibited NA enzymatic activity by a similar mechanism, where its CDR H3 loop adopts a protruding conformation with a tip that inserts into the NA catalytic site [69]. Using the same mechanism, mAbs that target influenza B virus (IBV) NA isolated from IBV-infected patients were able to inhibit NA enzymatic activity and protect against lethal IBV infection in mice in prophylactic and therapeutic settings [62] (Figure 2E). A recent study used a panel of N2 mAbs and generated escape mutant viruses to determine essential epitopes for mAb binding. They found that mutations at K199 and E258 had the largest impact on mAb binding and NA inhibition activity [70]. A similar study was performed using N1 mAbs, and escape mutations were found on the head domain of the N1 protein near the enzymatic sites S364N, N369T, and R430Q. Escape mutants were also detected on the sides and bottom of the NA (N88D, N270D, and Q313K/R) [71]. Another group generated a panel of mouse N1 mAbs and identified amino acids essential for mAb binding to the NA of a recent seasonal H1N1 virus, A/Brisbane/59/2007. They found that residues 273, 338, and 339 are conserved among NAs of seasonal H1N1 and the 1918 and 2009 pandemic H1N1 viruses, as well as H5N1 viruses [72]. mAbs targeting these three conserved residues effectively protected mice from heterologous lethal infection [72]. Overall, this information greatly increases our knowledge of neuraminidase and its essential epitopes, and it informs the development of influenza vaccines. However, our understanding of NA epitopes is still limited. More studies need to be performed in order to further characterize and identify antigenic sites.

## 6. NA Universal Vaccine

Vaccination is the best approach available to prevent influenza virus infections. The most commonly used vaccine is the split inactivated influenza vaccine [73]. The quantity of NA is not standardized and/or not present in currently licensed influenza vaccines and can vary between manufacturers, while the amount of HA is standardized in all seasonal vaccines [57,74]. Several approaches towards the development of a universal or broadly reactive influenza virus vaccine have focused on eliciting antibodies targeting the HA surface protein, with less interest focused on the NA [75,76]. However, studies showed that NA-based vaccines could elicit broadly protective antibodies [6,44,49,62,77]. Mice vaccinated with recombinant N1 NA protein stabilized by replacing the N-terminal cytoplasmic domain, transmembrane, and extracellular stalk with the tetramerization domain from the measles virus phosphoprotein (N1-MPP) produced robust antibody responses and were protected from a lethal H1N1 challenge [44]. mAbs isolated from a human donor previously infected with H3N2 were found to be broadly protective whether used prophylactically or therapeutically in mice against viruses from groups 1 and 2 of influenza A viruses as well as some influenza B viruses [49]. Depending on the conserved epitopes, the breadth of NAI antibodies varied from subtype-specific to pan-influenza [66,72]. The identification of conserved epitopes on NA could provide a potential strategy for a broadly protective vaccine. An example of this is designing an epitope-based vaccine that would provide a target for B and T cells to elicit an immune response against conserved NA epitopes. Recent studies used this approach to develop a more broadly reactive immune response; this was done either by incorporating the conserved NA epitope into the HA head domain of an H1N1 virus, creating a chimeric antigen, or by developing a self-assembling nanoparticle containing the NA conserved epitope in addition to two universal CD4 T cell epitopes to improve the immune response [78,79]. The higher amino acid mutation rate of HA than NA limits the effectiveness of HA-based vaccines [80]. Therefore, use of an NA-based vaccine may potentially provide a longer-lasting vaccine antigen than current vaccination methods that rely primarily on immune responses against HA. Clinical symptoms, peak viral titers, and viral shedding are inversely correlated with NA-inhibiting antibody titers [54,55,56,81].

NA content in vaccines correlates with anti-NA antibody response rates [82,83]. Increasing the NA content of vaccines and the standardization of the NA antigen in the influenza virus vaccine may contribute to increased vaccine efficacy. The use of VLPs expressing NA provided cross-protection against heterologous challenges in mice and ferrets [84,85,86]. Virus-like particles (VLP) expressing N1 from influenza strain A/California/04/2009 (N1 VLP) induced cross-protection against antigenically different influenza viruses [86]. Mice vaccinated with N1 VLP were protected from infection with H1N1, H5N1, and H3N2 strains, had lower lung viral titers, and less inflammation in their lungs compared to mice vaccinated with split inactivated influenza vaccine [86]. Computationally optimized broadly reactive antigens (COBRAs) have been extensively studied as an approach towards a universal influenza vaccine for multiple HA subtypes and were shown to be successful in eliciting protective antibodies [87,88,89,90]. Further studies showed that NA-based COBRA vaccines can elicit protective antibodies against seasonal and pre-pandemic strains [77]. Vaccination with N1-I COBRA elicited antibodies with broad NAI activity across three NA genetic clades (N1.1, N1.2, and N1.3). Mice vaccinated with N1-I COBRA were protected from lethal infection against the A/California/07/2009 (clade N1.1), A/Brisbane/59/2007 (clade N1.2), A/Swine/North Carolina/154074/2015 (clade N1.3), and A/Vietnam/1203/2004 (clade N1.2) viruses [77]. Additionally, a recent study has reported that computationally engineered recombinant NA proteins containing consensus sequences show broad protection within the H1N1 subtype in mice [91]. Nucleic acid-based vaccines are also a potential strategy for an influenza vaccine [92]. This method depends on expressing the target antigen in vivo following vaccination with a plasmid encoding for the gene of interest [93]. Mice vaccinated with plasmid DNA encoding N1 from H1N1 virus A/New Caledonia/20/99 were protected from lethal challenge with H5N1 influenza virus [94]. DNA plasmid encoding N2 protein induced full protection against challenge with homologous and drifted H3N2 strains, but failed to protect against H1N1 challenge [95]. Additionally, an mRNA-based NA vaccine elicited protection against infection in mice after one vaccination [96]. These studies are providing important steps towards a universal vaccine. A universal influenza vaccine has been defined as at least 75% effective against symptomatic influenza virus infection caused by group 1 and group 2 IAVs, with the protection lasting over 1 year for all age groups [97]. In addition to NA-based vaccines, additional studies are currently testing different combinations of universal vaccine targets, including M2 proteins and HA-based vaccines.

## 7. Conclusions

Significant progress has been made on the path toward a universal influenza vaccine. Neuraminidase is currently a major focus among scientists to reach that goal. NA usually shows lower antigenic drift rates compared to HA. Anti-NA antibodies have been shown to be cross-protective after being challenged with different influenza strains. In addition to NA, there are other targets for a universal vaccine, including the M2 ion channel and the HA stem, as well as some HA head approaches [98,99]. Since antigenic drift for NA does not correspond with antigenic drift for HA, quantifying and standardizing the amount of NA along with HA in the seasonal influenza vaccine has the potential to improve the efficacy of the vaccine [100]. A better understanding of NA-based immunity and its mechanisms of action can contribute to the design of better, longer-lasting, and more broadly protective vaccines.

## Data Availability

Not applicable.
